# The Brazos Valley Medical Association

**Published:** 1899-12

**Authors:** I. P. Sessions, W. B. Briggs

**Affiliations:** President; Easterly, Texas; Secretary; Easterly, Texas


For Texas Medical Journal.

### The Brazos Valley Medical Association.

EASTERLY, TEXAS, November 20, 1899.

The Brazos Valley Medical Association was called to order at Hearne, Texas, at 11 a. m. November 14, by President I. P. Sessions.

The prayer by Rev. C. C. Garrett was an earnest and most glorious effort. He asked the blessings of Almighty God upon the members of the Association.

Mayor Brady delivered the address of welcome on the part of the citizens. He complimented the Association and congratulated them upon the fourth year of their existence.

Dr. L. M. Bassett delivered the welcome address on the part of the local physicians.

Dr. M. L. Langford responded to both addresses. The following papers were read and discussed: "The First Stage of Labor," by Dr. W. C. Taylor; "The Baby's First Week," by Dr. W. H. Cummings; "The Baby's First Year," by Dr. J. C. Van Nuys; "Malarial Hæmaturia," by Dr. M. L. Langford; "Erysipelas," by Dr. M. L. Langford; "Railroad and Gin Accidents, and when to Operate," by Dr. J. P. Oliver.

Report of cases by Dr. J. W. Hudson and others.

Appropriate resolutions were adopted on the deaths of Dr. J. A. Boyd of Thorndale, and Dr. S. Arnold of Rosebud.

The Association received six new members.

Caldwell was selected as the next place of meeting, May 8 and 9, 1900.

A resolution of thanks to the citizens, and especially to the ladies, was passed by a rising vote.

The banquet was grand and joy reigned supreme.

Many who had papers were detained at home by serious sickness in their families or among their patients.

There were forty-seven members in attendance.

I. P. SESSIONS, President.

W. B. BRIGGS, Secretary.

